# Investigation on Spontaneous Abortion and Human Papillomavirus Infection †

**DOI:** 10.3390/vaccines8030473

**Published:** 2020-08-25

**Authors:** Mauro Tognon, Andrea Tagliapietra, Federica Magagnoli, Chiara Mazziotta, Lucia Oton-Gonzalez, Carmen Lanzillotti, Fortunato Vesce, Carlo Contini, John Charles Rotondo, Fernanda Martini

**Affiliations:** Department of Medical Sciences, University of Ferrara, Fossato di Mortara street, 64, 44121 Ferrara, Italy; tgm@unife.it (M.T.); tglndr@unife.it (A.T.); federica.magagnoli@student.unife.it (F.M.); mzzchr@unife.it (C.M.); tnglcu@unife.it (L.O.-G.); lnzcmn@unife.it (C.L.); ves@unife.it (F.V.); cnc@unife.it (C.C.)

**Keywords:** human papillomavirus, HPV, infection, spontaneous abortion, quadrivalent HPV vaccine, 9vHPV vaccine, 9-valent HPV vaccine, chorionic villi, PBMC, droplet digital PCR, voluntary interruption of pregnancy, antibody, ELISA

## Abstract

Viral infections are considered to be risk factors for spontaneous abortion (SA). Conflicting results have been reported on the association between Human Papillomavirus (HPV) and SA. HPV DNA was investigated in matched chorionic villi tissues and peripheral blood mononuclear cells (PBMCs) from women who experienced SA (*n* = 80, cases) and women who underwent a voluntary interruption of pregnancy (VI; *n* = 80, controls) by qualitative PCR and quantitative droplet digital PCR (ddPCR). Viral genotyping was performed using real-time PCR in HPV-positive samples. Specific IgG antibodies against HPV16 were investigated in sera from SA (*n* = 80) and VI (*n* = 80) females using indirect ELISA assays. None of the DNA samples from SA subjects was HPV-positive (0/80), whilst HPV DNA was detected in 2.5% of VI women (*p* > 0.05), with a mean viral DNA load of 7.12 copy/cell. VI samples (*n* = 2) were found to be positive for the HPV45 genotype. The ddPCR assay revealed a higher number of HPV-positive samples. HPV DNA was detected in 3.7% and 5% of SA and VI chorionic tissues, respectively, with mean viral DNA loads of 0.13 copy/cell in SA and 1.79 copy/cell in VI (*p* >0.05) samples. All DNA samples from the PBMCs of SA and VI females tested HPV-negative by both PCR and ddPCR. The overall prevalence of serum anti-HPV16 IgG antibodies was 37.5% in SA and 30% in VI (*p* > 0.05) women. For the first time, HPV DNA was detected and quantitatively analyzed using ddPCR in chorionic villi tissues and PBMCs from SA and VI women. Circulating IgG antibodies against HPV16 were detected in sera from SA and VI females. Our results suggest that HPV infection in chorionic villi may be a rare event. Accordingly, it is likely that HPV has no significant role in SA.

## 1. Introduction

Spontaneous abortion (SA) is the unintentional loss of the embryo or fetus before the 20th week of gestation [1]. Spontaneous embryo/fetus loss is considered the most common adverse complication during pregnancy [2,3,4]. The estimated SA occurrence, which varies according to the study population in question, is about 12–15% of clinical gestations [5]. Nearly 80% of SAs occur in the first trimester of pregnancy (first 12 weeks) [6], whereas 30% of pregnancies are lost between implantation and the sixth week [5]. Furthermore, the risk of SA occurrence directly increases with age, from 10–25% up to 60–70% in pregnant females under 25 years old and over 40 years of age, respectively [7].

Factors affecting SA have not been completely elucidated [8,9]. Genetic abnormalities, including chromosomal alterations and/or abnormal chromosomal numbers, are accountable for about half of SA events [10,11,12]. SA has also been associated with a large number of other factors, including ethnic origin, stress, occupational/chemical exposures, and lifestyle factors, such as obesity, smoking, and alcohol [13,14,15]. Other causes, which have been found to be related to SA include anatomical, endocrine/hormonal, and autoimmune/immunological abnormalities, as well as male factors [16,17]. Notably, up to 50% of SA cases lack a clearly defined etiology. Thus, they are considered idiopathic [5].

The relationship between SA and pathogenic infection agents is yet to be fully elucidated [2,3,4,18]. Infectious agents are considered SA risk factors, being potentially involved in about 40% of SA events [18,19,20]. Such infectious agents include viruses which can negatively impact pregnancy, being linked to miscarriage and other outcomes such as stillbirth and preterm delivery [18,21]. A number of human viruses, including herpes simplex viruses 1 and 2, cytomegalovirus, dengue virus, zika virus, adenovirus, and adeno-associated virus can potentially infect the placenta, the trophoblast, and/or the cytotrophoblasts after viremia or an ascendant infection [18,22,23,24,25,26,27]. It is known that cytomegalovirus and herpes simplex viruses can cross the placental barrier, thereby resulting in either SA events or severe birth defects, such as microcephaly [21]. Recent investigations into the association between SA and polyomaviruses (PyV), such as JC (JCPyV), BK (BKPyV), Merkel cell polyomavirus (MCPyV), have reported negative data. Indeed, PyV DNA has been detected with low prevalence in pregnant females and females who had SA [3,4,28].

Previous studies suggest that the human papillomavirus (HPV) may affect pregnancy outcome [18,29,30]. HPV is a DNA virus which includes a group of over 220 different types [31,32]. Based on their oncogenic potential, HPVs can be divided into two distinct groups, namely, (i) high-risk HPVs (HR-HPVs) and (ii) low-risk HPVs (LR-HPVs). HR-HPVs, including HPV16–18, are the most significant oncogenic viruses associated with the onset/progression of anogenital and upper respiratory tract tumors [33,34,35]. The nonavalent HPV vaccine (9vHPV) is available nowadays and protects against nine HR-HPV genotypes, i.e., HPV16/18/31/33/45/52/58 [36,37]. Currently, HPVs can be detected by qualitative Polymerase Chain Reaction (PCR) in uterine cervical specimens to determine specific viral genotypes [38], following a positive Papanicolaou (PAP) test. However, a more analytical assay, such as droplet digital PCR (ddPCR), for detecting/quantifying HPV DNA, has been recently established as a potential clinical diagnostic tool [39]. 

HPVs are responsible for one of the most common sexually transmitted viral infection among reproductive-age males and females worldwide [40,41]. These viruses have been detected in cytological samples derived from normal females worldwide, with a prevalence of about 12% [42]. In addition, immunological data indicate that approximately 60% of sera from females carry anti-HPV antibodies [43]. Recent evidence suggests that HPV may also affect fertility, the clinical pregnancy rate of medically assisted reproductive technologies (MARs), and pregnancy outcome [44,45,46,47,48]. For instance, the presence of HPV in semen samples from infertile couples can affect sperm quality parameters [49]. In addition, HPV DNA has also been detected in sperm fluids from males in miscarriage-affected couples [50]. 

Previous studies on SA and HPV infection reported conflicting results [24,51,52]. Indeed, HPV DNA sequences have been detected with a large range of prevalence, from 4% to 75%, in SA samples and in 20–24% of specimens from females who underwent voluntary interruption of pregnancy (VI) [24,51,52]. In addition, the majority of these studies only reported qualitative data [24,51,52]. These conflicting results have raised the question of whether HPV infection is linked to SA.

To this aim, herein the association between SA and HPV infection was evaluated. Specifically, qualitative and quantitative PCR methods were employed to analyze DNA derived from aborted tissues, i.e., chorionic villi as well as peripheral blood mononuclear cells (PBMCs) from SA females (cases) and VI females, employed as a control. In addition, the presence of IgG antibodies against HPV16 was investigated in sera collected from SA and VI females.

## 2. Methods

### 2.1. Samples

Samples were collected from two different cohorts of pregnant women, i.e., females who experienced spontaneous abortion (SA, *n* = 80, the cases) and females who underwent voluntary interruption of pregnancy (VI, *n* = 80), employed as a control. Chorionic villi tissue specimens (*n* = 160) and corresponding PBMCs (*n* = 160) and serum samples (*n* = 160) from SA (*n* = 80) and VI (*n* = 80) groups were taken from our sample collection [2,3,4]. Chorionic villi and blood samples were collected within 12 h from the abortion. Serum samples were isolated as reported [53]. All chorionic villi samples were obtained by expert gynecologists using standard procedures and manually selected from the aborted material, using sterilized scissors/scalpels. In SA and VI groups, the exclusion criteria were: (i) positivity for infections, such as presence of human immunodeficiency viruses (HIV), hepatitis B virus (HBV), hepatitis C virus (HCV), and syphilis; (ii) the presence of congenital/acquired immune deficiency syndrome/diseases; (iii) immunosuppressive therapies, which are well-known causes of spontaneous abortions; (iv) genetic diseases; (v) severe uterine or hormonal dysregulation; (vi) use of teratogenic drugs. The inclusion criteria were: (i) patient in the 18–42 age range; (ii) gestational age within the first 12 weeks; (iii) for the cohort of VI pregnancy, females selected according to Italian law, Bill 194, Article 6, and Comma B. The mean ages (± standard deviation) of SA and VI groups were 35 ± 4 and 31 ± 5, respectively. Written informed consent was obtained from all females. The study was approved by the County Ethics Committee of Ferrara, Italy, ID number 151078. Clinical samples were collected by Dr. Roberta Capucci and Dr. Alice Poggi, Obstetrics and Gynecology Clinic, University Hospital of Ferrara, as reported earlier [3,4].

### 2.2. DNA Isolation 

DNA was isolated and purified from chorionic villi specimens as reported [4]. Briefly, chorionic tissues (~25 mg/specimen) were incubated overnight with proteinase K at 56 °C to allow tissue digestion. Then, DNA was isolated using QIAmp DNA Blood and Tissue Extraction Kit (Qiagen, Milan, Italy) according to the manufacturer’s instructions [54]. PBMCs and serum samples were isolated from the peripheral blood by density gradient using Histopaque-1077 (Sigma-Aldrich, Milan Italy) [3]. Serum samples were then stored at −80 °C until the time of analysis. DNA from PBMCs was isolated with the QIAmp DNA Mini Extraction Kit (Qiagen, Milan, Italy). Each isolated DNA sample was quantified and evaluated for PCR suitability by spectrophotometric reading (NanoDrop 2000, Thermo Scientific, Monza, Italy) [55] and by amplifying the β-globin gene sequence, respectively [33]. Tight precautions were taken to avoid cross-contamination during DNA isolation procedures and PCR reactions [56]. In detail, DNA was purified simultaneously with a sample of salmon sperm DNA and a mock sample lacking DNA (distilled water) [57,58] and then subjected to PCR. DNA was stored at −80 °C until the time of analysis.

### 2.3. HPV DNA Detection

In the first phase of our analysis, the presence of HPV DNA in SA and VI chorionic villi and PBMCs was investigated by qualitative PCR, by amplifying the highly conserved HPV L1 genomic region, using the broad-spectrum HPV-specific GP5+/6+ primers set [31]. These primers can detect HPV16/18/6/11/31/33/45 genomes. Each PCR reaction was conducted with the recombinant plasmid pUC19 containing the HPV16 genome, used as a positive control. PCR reactions were carried out with 500 ng of human genomic DNA. Amplicons were analyzed using 2–2.5% gel electrophoresis.

### 2.4. HPV DNA Genotyping and Viral DNA Load Determination by qPCR

In the second phase, HPV genotyping and HPV DNA load determination were performed in HPV-positive samples, by quantitative PCR (qPCR), with the CFX96 Touch™ RT-PCR Detection System (Bio-Rad, Segrate, Milan, Italy). Briefly, qPCR was carried out in 10 μL; each reaction included 2× of the SsoAdvanced Universal SYBR Green Supermix, Bio-Rad (Hercules, CA, USA) and 0.5 μM of each primer. qPCR conditions were: 95 °C, 5 min and 45 cycles of 95 °C, 15 s, 60 °C, 30 s. Different type-specific primer sets targeting HPV16/18/31/33/45 genotypes were employed, as reported [59,60]. In addition, the presence of HPV6/11 genotypes was assessed using the following primer sets: HPV-6-F, 5′-GGGAACGCAGGTAGAGAAAC-3′, HPV-6-F, 5′-CTCCCGTACACTGTTTGTGG-3′, HPV-11-F, GCAACGCAGGTAGAGAAACAT and HPV-11-R, CTCTCGGGTGCTGTCGTCTA. Quantitative determination of HPV DNA copy number in the samples was performed with the GP5+/6+ primer set [31]. Briefly, recombinant plasmids containing HPV16/18/6/11/31/33/45 DNA were used as positive controls [59]. The *EIF2C1* gene was used as a housekeeping gene to determine the human cell equivalent of each sample. HPV DNA loads were reported as viral copies per human cell equivalent (copy/cell). Multiple negative controls, consisting in two DNA extraction controls containing salmon sperm DNA and a mock sample lacking DNA (distilled water), as well as an additional technical negative control containing sterile water, were included in each PCR/qPCR reaction. The samples were analyzed three times, without different results. 

### 2.5. Viral DNA Load Determination by ddPCR

HPV DNA load was determined using the specific ddPCR assay [39]. This quantitative method, which allows viral DNA sequences to be detected, was performed using the QX200 Droplet Digital PCR System-Bio-Rad (Bio-Rad, Segrate, Italy). DdPCR enables the HPV DNA load to be analyzed without an internal positive control, as it specifically provides an absolute quantification of viral DNA [39]. The ddPCR reaction contained 11 μL of a 2 ×ddPCR super mix (QX200 EvaGreen ddPCR, Bio-Rad, Segrate, Italy), 0.5 μL of each primer (final concentration 225 nM each), and 10 μL of DNA/ddH_2_O (~100 ng per reaction). Broad-spectrum HPV-specific GP5+/6+ primers were employed. The mixture was added to the DG8 cartridge with 20 μL of droplet formation oil, using an automated droplet generator (Bio-Rad, Segrate, Italy). Every sample was partitioned into ~20,000 droplets, which were then transferred into a 96-well PCR plate, covered with pierceable foil, heat-sealed using a PX1 PCR Plate Sealer (Bio-Rad, Segrate, Italy), and placed in a thermal cycler (SimpliAmp, Applied Biosystem, Milan, Italy). Cycling conditions were as follows: heat to 95 °C for 5 min, followed by 94 °C for 30 s, 48 °C and for 1 min for a total of 45 cycles, then 5 min at 90 °C, ending at 4 °C. After PCR, the 96-well PCR plate was placed in the reader [39]. Data were analyzed using with the QuantaSoft analysis tool (Bio-Rad, Segrate, Italy). The cellular eukaryotic translation initiation factor *2C1* gene (Bio-Rad, Segrate, Italy), located at chromosome 1p34.3, was used as a housekeeping gene to determine the human cell equivalents of each sample under analysis. Each ddPCR experiment included two DNA extraction negative controls and an additional negative control (H_2_O) without DNA. 

### 2.6. Determination of Serum IgG Antibodies against HPV16 

Specific IgG antibody levels against HPV16 L1, which is the main capsid viral protein, were qualitatively determined in serum samples using the enzyme-linked immunosorbent assay (ELISA) kit human papillomavirus type 16 L1-capsids (HPV16L1) antibody (IgG) (Cusabio, Clinisciences, Milan, Italy). All reagents were employed at room temperature. Serum samples were diluted 1:1000 and thoroughly mixed before use. The plate with dispensed samples was read by optical density (OD) with the spectrophotometer at 450 nm wavelength (Thermo Electron Corporation, model Multiskan EX, Vantaa, Finland). The cut-off value was calculated according to the manufacturer’s instructions. Samples were considered HPV-positive when the ratio between the OD of each sample and the OD of negative controls was ≥2.1. Samples with optical density below the cut-off ratio value were considered negative.

### 2.7. Statistical Analysis

A two-sided chi-square test was employed to statistically analyze viral DNA prevalence in SA and VI chorionic tissues and PBMCs. Viral load values and ELISA OD values were analyzed with the D’Agostino–Pearson test for normality, and means were compared to the non-parametric Mann–Whitney U test [61]. Statistical analyses were carried out using the Graph Pad Prism version 5.0 for Windows (Graph Pad, La Jolla, CA, USA) [62]; *p*-values < 0.05 were considered statistically significant.

## 3. Results

### 3.1. HPV DNA Detection, Genotyping, and Viral DNA Load Quantification

HPV DNA sequences were investigated in 320 samples, including 80 chorionic villi and 80 PBMCs from SA females (*n* = 80) and 80 chorionic villi and 80 PBMCs from VI females (*n* = 80, employed as a control). DNA was investigated using the qualitative PCR technique. None of the DNAs from the SA samples tested positive for HPV (0/80), whereas HPV sequences were detected in 2/80 (2.5%) DNA samples from chorionic tissue specimens in the VI group (*p* > 0.05) (Table 1). Moreover, DNA from all the PBMC samples of SA and VI females tested HPV-negative (Table 1). 

Viral genotyping was performed with the qPCR method in HPV-positive VI specimens (*n* = 2) using different type-specific primer sets targeting HPV6/11/16/18/31/33/45 genomes. The results indicated that these two HPV-positive DNA samples from the VI group were both positive for the HPV45 genotype. 

HPV DNA load was determined by qPCR using broad-spectrum HPV-specific GP5+/6+ primers in the two HPV45-positive samples. Quantitative results indicated that the mean HPV DNA load detected by qPCR in chorionic tissues from the VI group (*n* = 2) was 7.12 copy/cell (range 14.13–0.11 copy/cell).

In the second phase of this study, the specific ddPCR assay for investigating/quantifying HPV DNA sequences was employed to analyze DNA from SA (*n* = 80) and VI (*n* = 80) chorionic villi as well as SA (*n* = 80) and VI (*n* = 80) PMBCs, for a total of 320 samples. Viral DNA was ddPCR-amplified employing the broad-spectrum HPV-specific GP5+/6+ primer set [39]. The ddPCR assay reveled a higher number of HPV-positive samples in both groups under analysis. Specifically, HPV DNA was detected in 3/80 (3.7%) DNA samples taken from chorionic tissues in the SA group and 4/80 (5%) VI samples (*p* > 0.05) (Table 1). Two VI females tested HPV-positive by both PCR and ddPCR assays. Viral genotyping was then extended to the other samples that tested HPV-positive by ddPCR, i.e., *n* = 3 SA and *n* = 2 VI. These samples tested HPV-negative in the type-specific analysis carried out by qPCRs.

**Figure 1:**
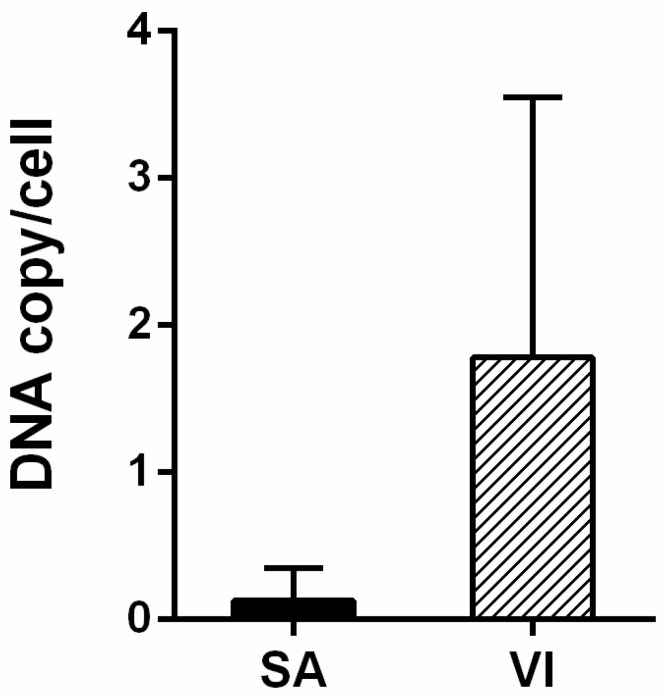
Mean HPV DNA load detected by ddPCR. Mean HPV DNA load (viral DNA copy/cell) was determined in HPV-positive chorionic villi specimens from SA (*n* = 3) and VI (*n* = 4) women. Error bars represent standard error of the mean (SEM). The difference in viral load between the SA and VI groups was not statistically significant (*p* > 0.05).

The mean HPV DNA load detected in chorionic tissues by ddPCR was 0.13 copy/cell (range 0.002–0.385 copy/cell) in SA samples (*n* = 3) and 1.786 copy/cell (range 0.004–7.086 copy/cell) in VI samples (*n* = 4) (Figure 1).

Differences in HPV DNA loads in SA and VI specimens were not statistically significant (*p* > 0.05). PBMC samples from both SA and VI cohorts tested HPV DNA-negative (Table 1).

### 3.2. HPV Antibody Detection by Indirect ELISA 

Serum samples from SA (*n* = 80) and VI (*n* = 80) cohorts were analyzed for IgG antibodies against the HPV L1 protein using an ELISA test. The overall prevalence of IgG antibodies reacting to the HPV16 L1 capsid protein in these sera was 37.5% (30/80) in SA and 30% (24/80) in VI (Table 2) samples. The difference between SA and VI groups was not statistically significant (*p* > 0.05). 

In SA and VI sera, the prevalence of IgG antibodies against HPV-16 L1 protein was as reported above. Statistical analyses revealed no significant differences in HPV-16 prevalence between SA and VI (*p* > 0.05) females. Statistical analysis was performed by the chi-square trend test.

**Figure 2:**
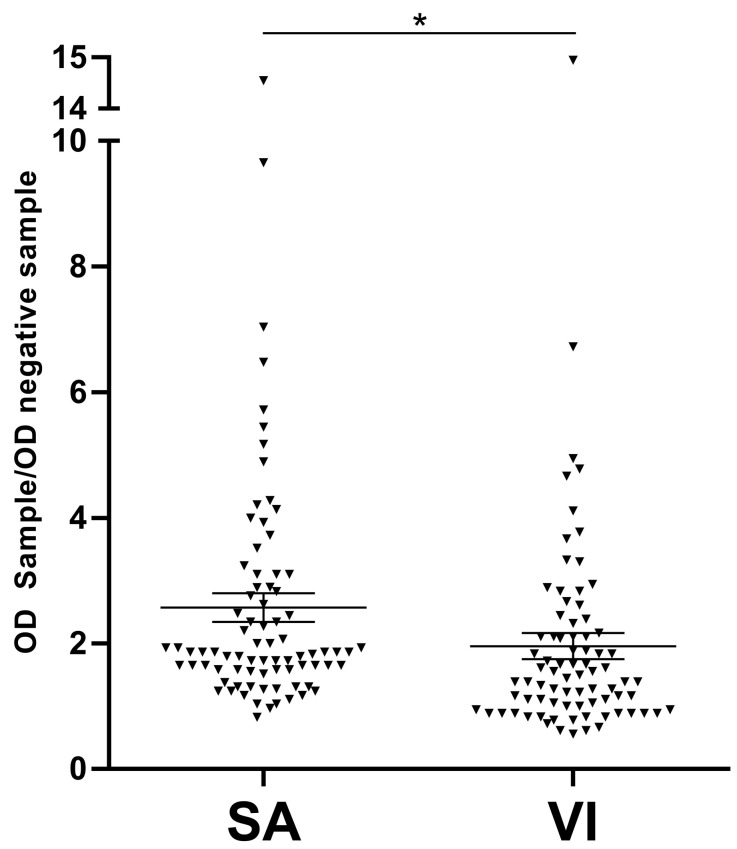
Serologic profiles of human serum antibody (IgG) reactivity to human papillomavirus type 16 (HPV-16) L1 capsid protein. Immunologic data are from sera of females who had spontaneous abortion (SA, *n* = 80) and females who underwent voluntary interruption of pregnancy (VI, *n* = 80). Results are presented as mean SA and VI OD sample/OD negative sample ratios. In this scatter dot plotting, each plot represents the dispersion of OD ratios to a mean level, indicated by the line inside the box with SEM (Standard Error of Mean) for the two groups of females analyzed. The difference of OD sample/OD negative sample ratio for reactivity with HPV16 L1 capsid protein is statistically significantly higher in sera from SA women compared to sera from VI women (* *p* < 0.05). Data were analyzed with Mann–Whitney U test; * *p* < 0.05.

Serum samples were considered HPV16-positive when the ratio between the OD of each sample and the OD of the negative controls was ≥2.1. In this investigation, the OD ratio differentiating HPV-negative sera (OD ratio ≤ 2.1) from HPV-positive sera (OD ratio ≥ 2.1) was 2.1. Immunological sera profiles of SA and VI females reacting to the HPV16 L1 antigen are shown in Figure 2. 

The mean OD values detected were 0.038 (± 0.029 [Standard Deviation of Mean]) and 0.034 (± 0.033 [SD]) in SA (*n* = 80) and VI (*n* = 80) samples, respectively. It is worth noting that the difference in the OD mean value for reactivity to the HPV16 L1 capsid protein was statistically significantly higher in sera from SA women compared to sera from VI women (*p* < 0.05).

The presence of anti-HPV16 antibodies was subsequently verified in SA (*n* = 3) and VI (*n* = 4) females testing HPV-positive in their chorionic villi. Out of *n* = 4 chorionic villi HPV-positive VI females, *n* = 2 HPV45-positive subjects carried serum antibodies against HPV16, while the remaining *n* = 2 had negative sera. In the SA group, out of *n* = 3 chorionic villi HPV-positive females, *n* = 2 carried anti-HPV16 antibodies in their sera, while *n* = 1 was HPV-negative. 

## 4. Discussions

SA represents the most common pregnancy adverse outcome. Viral infections are considered to be SA risk factors [18]. HPV is one of the most sexually transmitted viral infection among humans of reproductive age [40]. Herein, the association between SA and HPV infection was investigated. 

To establish whether HPV might be associated with SA, HPV DNA sequences were assayed in DNA from chorionic villi and PBMCs from SA and VI female cohorts. Chorionic villi were specifically chosen as they represent the major separating surface between mother and embryo/fetus, since viruses infecting PBMCs after maternal viremia can cross the placenta by hematogenous spread [18,63,64], inducing, in the worst cases, SA [21]. Moreover, HPV DNA was qualitatively/quantitatively investigated with different assays, employing broad-spectrum HPV-specific GP5+/6+ primers, which have been recently applied to ddPCR-based methods [39]. In this study, HPV DNA was detected by PCR/ddPCR at a low rate in SA and VI chorionic villi. Although 2.5% of VI was HPV-positive by both PCR and ddPCR assays, ddPCR detected HPV DNA in three and two additional SA and VI samples, respectively. These results indicate a higher analytical detection rate for ddPCR compared to the well-established qualitative PCR. At the same time, the ddPCR approach employed herein confirmed that this method is reliable in investigating HPV DNA in clinical samples [39], including chorionic villi. 

The results of the present study are consistent with previous works reporting low HPV rates, 4–7%, in chorionic villi [24,65,66], including trophoblastic [66,67] and Hofbauer cells [51], which are the outer layer cells of the blastocyst and the placental macrophages, respectively [51]. Furthermore, despite never being demonstrated in vivo [68,69,70], in vitro studies have indicated that HPV could replicate in trophoblast cells [68,69,70,71], suggesting that this virus may not be strictly keratinocyte-specific [68,69,70]. The low rates obtained in our study, in agreement with previous reports, indicate that HPV has low tropism for chorionic villi. However, the hypothesis that HPV can potentially infect pregnant females by crossing the placental barrier has been largely questioned. HPV DNA has been reported with high variable prevalence, 4–75%, in cord blood [66,72,73,74], amniotic fluids [75,76,77,78], cervical tissues [75,78,79,80], as well as fetal membranes, such as the placenta [51,66,75,78,79,81,82] and the decidua [24,83]. Other studies conducted on cord blood [75], amniotic fluids [75,77,84], and fetal membranes [79] do not support this evidence. This variability may depend on a large range of parameters, including HPV detection methods, differences in risk factors, such as other sexually transmitted infections, history of cervical dysplasia, genital warts, and maternal/gestational age [66,71,79,81]. 

In this study, in order to avoid confounding variables, we excluded females positive for (i) HIV, HBV, HCV, and syphilis infections; (ii) congenital/acquired immune deficiency syndromes or receiving immunosuppressive therapies; (iii) well-known causes of SA, i.e., genetic factors and anatomical/hormonal complications. SA and VI females were of similar ages, and samples were collected within the 12th gestation week. Another relevant aspect related to such conflicting results is that materials could be HPV-contaminated during sampling. Indeed, neonates can contract the HPV infection during passage through an HPV-infected vaginal canal [85], whilst a previous study has reported negative data on HPV infection in aborted tissues collected by transabdominal puncture, thereby bypassing the cervix (HPV-positive in 25% of samples) [79]. A low HPV rate has also been obtained by excluding pregnant females with HPV-positive uterine cervical lesions [75]. In this context, sample contamination in studies reporting high HPV rates cannot be ruled out [79]. In our study, in order to avoid contamination, sampling, DNA isolation, and PCR/qPCR/ddPCR assays underwent strict quality control.

In summary, previous studies reported conflicting results on the association between SA and HPV infection. Indeed, HPV DNA has been reported with a large range of prevalence, from 4% to 75%, in SA samples [24,51,52]. The reported conflicting data could be due to confounding variabilities, whilst the HPV DNA detection methods employed in these studies were mainly based on PCR assays, which provide qualitative detection of viral DNA. In the present investigation, we found 3.7% and 5% positivity for of HPV DNA in SA and VI chorionic villi, respectively, employing, for the first time, the ddPCR assay, which is a novel and highly analytical method for the detection of viral DNA. Our results indicate that ddPCR is a valuable technique in detecting/quantifying HPV DNA in clinical samples, including chorionic villi, and suggest that HPV infection in this tissue may be a rare event.

Quantitative data, obtained for the first time using both qPCR and ddPCR methods in DNA from chorionic villi, indicated a low HPV DNA load. In addition, none of the investigated PBMCs tested HPV DNA-positive, suggesting the absence of viremia. As the chorionic villi-HPV-positive SA and VI females had HPV-negative PBMCs, hematogenous transmission can be excluded [78]. Our results indicate that the SA females examined did not carry a higher HPV burden than the females undergoing VI and support the view that this viral agent plays no role in SA. 

The role of HPV infection in SA has been debated. Growing evidence indicates that HPVs play a role in male infertility, as its DNA has been found in the semen from infertile males [86,87]. HPV infection seems to be a predictive factor of negative pregnancy outcome [50,52]. Indeed, previous data have indicated a ratio of SA/VI HPV-positive aborted tissues of 2:1 [52], while HPV DNA has been detected in semen samples in a significant fraction of miscarriage-affected couples undergoing MARs [50]. The fertilization potential of HPV-positive males can be improved after HPV vaccination, with a demonstrated increase in pregnancy rate [48]. In addition, sperm-washing techniques employed to remove HPV from semen samples have been reported as improving MAR pregnancy rates [46,47]. However, a recent meta-analysis conducted on 18 studies excluded any association between HPV and pregnancy/SA rates [45], indicating that HPV does not affect pregnancy outcome after MAR [88]. The implication of HPV in negative pregnancy outcomes has also been excluded in a number of other works [24,89], including those reporting high HPV rates in SA tissues [51,81]. A previous study reported a lack of HPV infection in chorionic villi from miscarriage-affected females [65]. Accordingly, our data indicate no association between SA and HPV infection. In other words, HPV may infect the placenta [52] without having an impact on embryogenesis. 

Herein, two VI cases tested HPV45-positive, with HPV45 being a HR-HPV [66], while the remaining samples, found to be HPV-positive by ddPCR, were HPV-negative when analyzed by type-specific qPCR. These results indicate a higher analytical detection rate for ddPCR compared to qPCR [90]. Studies carried out on placental tissues detected HPV genotypes in aborted tissues, including HPV6/11/16/18/58/66/82/83 [24,66,81], while a case report of epidermodysplasia verruciformis also reported the presence of HPV3/5/8/24/36 [78]. Many studies have detected both LR- and HR-HPVs in cervical samples from pregnant females. In this study, it is plausible that the two VI females found to be HPV45-positive were administered the pre-pubertal quadrivalent vaccine, which is active against HPV6/11/16/18 [91,92], although data on cross-protection against non-vaccine HPV types have been reported [93,94]. It is important to point out, however, that the vaccination status of the two VI females found to be HPV45-positive is unknown. In addition, the two HPV45-positive VI females carried anti-HPV16 antibodies in their sera. It is also possible that these females naturally seroconverted upon HPV16 infection. Immunological tests, extended to SA and VI sera, failed to show any differences in prevalence between groups, although a higher titer was found in SA compared to VI women. Our data are in agreement with seroprevalence results reported earlier [95,96], indicating that HPV16 infects humans, including pregnant females, at a high rate. Since all the DNA samples in our study from SA chorionic villi/PBMC were HPV16-negative, the involvement of HPV in SA is unlikely.

## 5. Conclusions

In conclusion, this study suggests the lack of evidence of an association between SA and HPV infection. Few copies of HPV DNA were detected in chorionic villi from SA and VI females, while two VI females tested HPV45-positive. Negative data for PBMCs, which all tested HPV-negative, indicate the lack of an active infection. Immunological data confirmed that serum anti-HPV16 antibodies can be detected in pregnant females. Our results suggest that HPV infection in chorionic villi may be a rare event, whereas it is likely that HPV has no significant role in SA. 

This study, which contributes to elucidating the role of HPV during pregnancy, could be useful for those clinicians/operators working in the fields of gynecology/obstetrics and medically assisted reproduction.

## Figures and Tables

**Table 1:** Prevalence of human papillomavirus (HPV) DNA sequences in chorionic villi and peripheral blood mononuclear cells (PBMCs) samples from spontaneous abortion (SA) and voluntary interruption (VI) of pregnancy groups.

Groups Number of Positive Samples/Total of Samples (%)				
	PCR		ddPCR	
	Chorionic Villi	PBMCs	Chorionic Villi	PBMCs
SA	0/80 (0)	0/80 (0)	3/80 (3.7)	0/80 (0)
VI	2/80 (2.5)	0/80 (0)	4/80 (5)	0/80 (0)

In SA and VI chorionic villi and PBMCs, the prevalence of HPV DNA was as reported above. Statistical analyses revealed no significant differences in HPV prevalence between SA and VI (*p* > 0.05) females. Statistical analyses were performed by the chi-square trend test. PCR: Polymerase Chain Reaction; ddPCR: droplet digital PCR.

**Table 2:** Prevalence of IgG antibodies reacting to HPV-16 L1 capsid protein detected in sera from SA and VI groups.

Groups	Number of Sera	Median Age ± SD	Number of Positive Samples/Total of Samples (%)
SA	80	35 ± 4	30 (37.5)
VI	80	31 ± 5	24 (30)
